# Local History as a Relaxation for the Practitioner

**Published:** 1910-08-13

**Authors:** 


### LOCAL HISTORY AS A RELAXATION FOR THE PRACTITIONER.

One of the most absorbing hobbies that the doctor, if practising in the provinces, can take up is the compilation of the history of the town or parish in which his lot is cast. It is certainly a pursuit likely to ameliorate that sentence of penal servitude for life to which fate, chance, or the force of circumstances has condemned him; for while it turns his thoughts back to a past full of interest and distracts his mind from the worries of daily life, it is so elusive and protracted a pursuit that it will occupy a lengthy space of spare time to write of almost any parish a history that could pretend to be comprehensive. But it is not an inexpensive pursuit, and anyone who finds its fascination fasten upon him must be prepared to spend not a little on railway fares and many hours of absence from his practice. For, as Dr. Cox says, in his little brochure, "How to write the History of a Parish," most of the history of his locality must be sought in London, where it is stored in a great variety of places, such as the Record Office, the British Museum Library, Somerset House, Lambeth Palace Library, and other repositories of books and manuscripts. Farther afield still is the Bodleian at Oxford, the most valuable library outside London, while nearer home are the Cathedral muniments of his diocese with its episcopal registers and the records in the local Registry of Wills.

Since the most interesting periods of local history are the most remote, and therefore anterior to the invention of printing, the would-be historian must master the art of deciphering ancient manuscripts—by no means an easy one, though fascinating to an unexpected degree. Possibly some have an innate talent for acquiring this needful art of interpreting the intricacies of the scripts of scribes of olden time, but there are few to whom long and patient study will not be found a *sine quâ non* to facility and sureness in its practice. There are several books published on this subject, from the excellent "Court-hand Restored" of the eighteenth century to Thoyt's "How to Decipher Old Manuscripts," and the little book by Dr. Cox aforesaid. The first has the advantage of many facsimile reproductions of old alphabets and portions of ancient manuscripts progressively arranged from the time of the Conquest onwards. Nowadays courses of instruction in Palæography are held by the London University—perchance by others—though it is hardly possible a doctor would be able to avail himself of them. His best course therefore would be to study at his leisure such original documents and reproductions in facsimile as he can obtain. The various old folios published by the Record Commission contain specimen pages of the original manuscripts, with full extensions. Among them may be named the Calendars of the Patent, Charter, and Originalia Rolls, issued at the beginning of the last century, while facsimiles of Domesday and other ancient MSS. are also procurable. The first necessary steps are to study the different forms that various letters of the alphabet assumed in the course of ages, and to familiarise oneself with the contractions which it became needful to adopt in order to expedite the tedious process of handwriting. For caligraphy varied much with succeeding centuries, and it is by no means always the most ancient documents that present the greatest difficulties in deciphering, so that many scripts of the Plantagenet period are perspicuously plain compared with the handwriting of Elizabethan times. It is the inability to read ancient manuscripts that has caused so many local histories to commence with post-Reformation times, to the great loss of much interesting matter and of all pretence to comprehensiveness; and thus so many of these compilations are based on parish registers, heraldic visitations, and churchyard inscriptions, while Domesday, episcopal registers, and the long series of what are called public records are left unexplored. As these include a vast range of subjects and carry the history of many an obscure parish and its families back to very early times the most interesting and illuminating details of the past are missed, and a very partial presentment of local circumstances and affairs results.

It is important, too, to know that much of the history of a parish, village, or small town centres round the church or churches, and the manor or manors that composed or contained it. In connection with the first may be found records of its foundation, or dedication, its endowment deeds, and its registers, as well as the story enshrined in its stones and fabric, and in its inscriptions within and without. In addition the tithe maps, if ancient, will be found to contain many particulars concerning such things as field- and place-names and other items throwing light upon the past. As regards the manor there may be still extant, either in private or public possession, its court rolls and kindred documents, some so numerous that they might extend to miles in length, and containing much interesting and quaint detail of persons, places, and things.

With the history of a parish, of course, is intimately interwoven that of the chief families who lived and moved and had their being in the neighbourhood in question. The records of such must be sought in the sources aforesaid, and in addition and more in particular in Heralds' Visitations, "Proofs of Age," registries of wills, and old county histories.

